# Cryopreservation the seeds of a Taiwanese terrestrial orchid, *Bletilla formosana* (Hayata) Schltr. by vitrification

**DOI:** 10.1186/1999-3110-54-33

**Published:** 2013-09-12

**Authors:** Wei Hsin Hu, Yue Han Yang, Song Iuan Liaw, Chen Chang

**Affiliations:** 1 Department of Biology, National Museum of Natural Science, Taichung, 404 Taiwan; 2 Department of Horticulture, National Chung Hsing University, Taichung, 402 Taiwan; 3 Department of Life Sciences, National Chung Hsing University, Taichung, 402 Taiwan

**Keywords:** Germination, Loading, PVS2, Viability, Vitrification

## Abstract

**Background:**

The cryopreservation of orchid seeds is an important conservation method, studies of the effects of cryopreservation on the seeds of wild orchids are scant. This investigation was to establish a method for the vitrification and cryopreservation of seeds of *B. formosana* that may be suitable for the long term storage of Taiwan native orchid germplasm for conservation purposes.

**Results:**

The germination rate and morphological stability of seeds from spontaneous-dehiscent capsules of *Bletilla formosana* (Hayata) Schltr. were evaluated after cryopreservation by vitrification. The germination rates of cryopreserved seeds varied according to immersion time and the vitrification method used. Seeds that were dehydrated by immersion in loading solution (LS; 2.0 M glycerol, 0.4 M sucrose) for 10 min to 30 min then transferred to plant vitrification solution 2 (PVS2) for 30 min prior to freezing in liquid nitrogen (LN) showed significantly higher germination rates than seeds immersed in PVS2 only. The optimal immersion times were 10 min for LS and 30 min for PVS2, resulting in an *in vitro* germination rate of 91%. Germination was not observed for cryopreserved seeds that were dehydrated by immersion in LS only. Seed viabilities and germination rates did not vary significantly for cryostorage times from 10 minutes to 1 year.

**Conclusions:**

This study improve, an efficient protocol was established that maintained seed viability and enhanced the germination rates of seeds, compared with previously described cryopreservation methods, and the germinated seeds showed normal morphology of both vegetative and reproductive organs.

**Electronic supplementary material:**

The online version of this article (doi:10.1186/1999-3110-54-33) contains supplementary material, which is available to authorized users.

## Background

There are many techniques available for the conservation of plant genetic resources of endangered species. These include *in-situ* and *ex-situ* conservation practices, micropropagation, seed germination, regeneration from explants, and cryopreservation (Nitzsche, 1983; Rick, 1984; Stanilova et al., 1994; Chang et al., 2000). The cryopreservation of wild orchid seeds is an important conservation method. Seeds are heterogeneous in most wild orchids, and preservation of seeds can help conserve the genetic diversity of plant populations. Compared with traditional methods of storage, cryopreservation is cost-effective, requires little space, and is efficacious for the long-term storage of plant genetic resources (Engelmann, 2011). Therefore, seed cryopreservation is an effective and powerful tool for *ex-situ* conservation (Thammasiri and Soamkul, 2007; Hirano et al., 2009).

Though many reports of the cryopreservation of orchids exist in the literature, studies of the effects of cryopreservation on the seeds of wild orchids are scant (Pritchard, 1984; Popova et al., 2003; Hirano et al., [Bibr CR9][Bibr CR10][Bibr CR8]). Vitrification is a simple, fast, and effective method for cryopreservation. It eliminates the need for controlled slow freezing, and permits cells and meristems to be cryopreserved by direct transfer into liquid nitrogen (LN) (Thammasiri and Soamkul, 2007; Tsai et al., 2009).

*Bletilla formosana* (Hayata) Schltr. is a species of terrestrial orchid that is native to Taiwan and southern China. It is found on seashore cliffs, lowland river banks, and mountain slopes (Su, 2000). Previous studies have shown that germination rates of *B. formosana* decreased to 47-57% after storage at 3°C of 6 months (Chang et al., 2006). The dry pseudobulbs of the closely-related *B. striata* are widely used in Chinese traditional medicine (Lin et al., 1994; Chen et al., 2009). The long term storage methods developed for the seeds of *Bletilla* may also be useful for the conservation of other orchid species of the same genus (Ishikawa et al., 1997).

The aim of our investigation was to establish a method for the vitrification and cryopreservation of seeds of *B. formosana* that would result in increased viability, compared with conventional cryostorage methods. An analysis of potential detrimental effects on the future development of the seedlings from cryopreserved *B. formosana* seeds was also performed. Such a method of seed cryopreservation may be suitable for the long term storage of Taiwan native orchid germplasm for conservation purposes.

## Methods

### Plant materials

Mature seeds from dehiscent capsules of *B. formosana* were collected from Puli Branch Station of the Taichung District Agricultural Research and Extension Station in Nantou, Taiwan. The seeds were stored in screw cap tubes (SCT-50MC-R-S) with silica gel at 4°C.

### Osmoprotection and cryopreservation of seeds

For loading treatments, approximately 2000–3000 seeds were transferred to 2.0 ml plastic cryotubes with 1.0 ml loading solution (LS, Matsumoto et al., 1994) containing 2 M glycerol and 0.4 M sucrose in 1/2 Murashiage and Skoog basal medium (MS, Murashige and Skoog, 1962) at room temperature (27 ± 2°C) for different periods of 0, 10, 20, or 30 min. In order to separate the floating seeds from the LS in the cryotube, make sure that the tip of the pipet touches the bottom of the cryotube before draining the LS out. After the LS was removed, the seeds were dehydrated on ice for 30 min with 1.0 ml plant vitrification solution 2 (PVS2, Sakai et al., 1990) containing 30% glycerol, 15% ethylene glycol, 15% dimethyl sulfoxide in 1/2 MS basal medium supplemented with 0.4 M sucrose. The pH of all media was adjusted to 5.8 with 0.1 M NaOH before autoclaving at 121°C for 15 min, 117.7 kPa. The PVS2 was replaced with 0.5 ml fresh solution after upon treatment, and the seeds in the cryotubes were directly plunged into LN for 30 min. The cryotubes were rapidly re-warmed in a 40°C water bath for 1 min. About 0.5 ml of 1/2 MS medium supplemented with 1.2 M sucrose was added to each cryotube, and the tubes were incubated for 10 min at room temperature. The liquid was decanted from the cryotubes, and 1 ml of fresh 1/2 MS medium supplemented with 1.2 M sucrose was added. The tubes were then incubated for 30 min at room temperature.

For vitrification treatments, seeds in cryotubes were immersed in LS for 30 min, and then immersed in PVS2 for 0, 10, 20, 30, or 120 min. Other conditions were identical to the loading treatments.

For cryopreservation duration, all procedures and conditions were same as in above- mentioned loading treatment except that seeds in cryotubes were immersed to LS for 30 min combined with PVS2 30 min, and then plunged into LN for 0, 10, 20, 30 min, 1 week, or 1 year.

### Seed germination

After completing all the cryopreservation treatments, seeds from each treatment were surface-sterilized in a lamina flow cabinet with 1% sodium hypochlorite and one drop of Tween-20 for 10 min, prior to rinsing three times with sterile distilled water. The aseptic seeds were then sown in 20 × 150 mm test tubes (Pyrex, No. 9820) with 8 ml of 1/2 MS salts modified medium (SD medium) supplemented with 0.5 mg/l niacin, 0.5 mg/l pyridoxine HCl, 0.1 mg/l thiamine HCl, 100 mg/l myo-inositol, 15% coconut milk, 0.6% potato powder (PhyotoTechnology Laboratories, Shawnee Mission, KS), 0.1% peptone, 2% sucrose and 0.8% agar (Medray Biotech, Taiwan) (pH 5.8). Seeds were maintained at 25 ± 1°C for 12 h photoperiods with the light intensity of 44.5 μmol m^-2^s^-1^ (daylight fluorescent tubes FL-30D/29, 40 w, China Electric Co, Taipei, Taiwan). After 4 weeks, the number of germinations was counted by observation under a stereomicroscope. Seed germination was defined as embryo swelling the coat and turning green. Germination rate was calculated as the ratio of germinations to the total number of seeds sown. 200–300 seeds were recorded per replicates. Values are means of 5 replicates.

### TTC staining for viability assessment

The viability of the seeds following each treatment was evaluated using the 2,3,5-triphenyltetrazolium chloride (TTC) test. Seeds were incubated in 1% TTC solution for 1 day at 27 ± 2°C in the dark. The number of embryos stained by TTC was counted, and the percentage of TTC-stained seeds represented the survival rate.

### Statistical analysis

The survival rate and germination rate of the seeds were analyzed by using ANOVA. Means were compared using Duncan’s Multiple Range Test at α = 0.05.

### In vitro seedling culture and transplanting

Eight-week-old protocorms were transferred to flasks containing 100 ml of SD medium. After three rounds of sub-culture in SD medium at 8-week intervals, plantlets 4–6 cm tall were transplanted in 5 cm diameter plastic pots with 1:1 (v/v) peat moss and fern chips, and grown in a pan and fan greenhouse.

## Results

**Figure 1:**
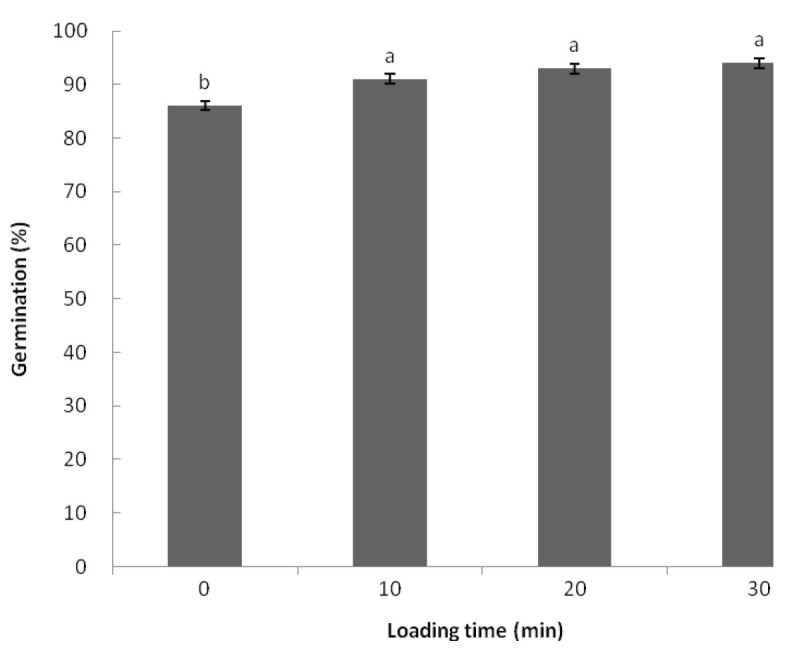
**Effect of loading solution treatment on the germination rates of** ***B. formosana*** **cryopreserved seeds.** After loading treatments, seeds were immersed in PVS2 for 30 min and cryopreserved for 30 min. Values are means of 5 replicates (200–300 seeds per replicate). Germination percentage was calculated after 4 weeks of culture.

The mature seeds of *B. formosana* are minute and dust-like in appearance, they measure approximately 900 μm × 150 μm and are fusiform in shape. In our study, seeds sown *in vitro* had an average germination rate of 78% (data not shown). Seeds treated with loading solution for 0, 10, 20, or 30 minutes before cryopreservation had *in vitro* germination rates of 86%, 91%, 93%, and 94%, respectively, after 4 weeks of culture (Figure 1). LS treatment increased seed germination rates, but no statistically significant difference in germination rates resulting from the various exposure times was observed.

The optimal dehydration time with PVS2 after 30 min of loading was determined. No germination was observed without PVS2 treatment. Seeds treated with PVS2 for 10 min had TTC staining of 85% (Table 1). Seeds treated more than 20 min had over 90% TTC staining, with germination rates of 69-78%. The highest germination rate was obtained after dehydration with PVS2 for 10 min.

**Table 1:** **Effects of PVS2 treatments on the TTC staining and the germination rate of** ***B. formosana*** **cryopreserved seeds**

Seed	PVS2 treatment period^1^				
	0 min	10 min	20 min	30 min	120 min
TTC staining (%)^2^	0	85	93	95	94
Germination rate (%)^3^	0^c^	78^a^	69^b^	72^ab^	69^b^

^1^Seeds were immersed in loading solution for 30 min and cryopreserved for 30 min.

^2^Approximately 300–350 seeds were tested in each TTC staining.

^3^Values are means of 5 replicates (200–300 seeds per replicate). Means followed by the same letters are not significantly different by Duncan’s test (*P*=0.05). Germination rates were calculated after 4 weeks of culture.

For cryopreservation, the seeds stored in LN for one week showed 96% TTC staining, and seeds with no LN treatment showed 77% staining (Table 2). The *in vitro* germination rates from seeds treated with LN for 10 min to 1 year were 83.7-90.1%. Germination rates for all treatments were significantly higher than non-LN treated seeds (79%). The highest germination rate of 90.1% was obtained from LN storage for 1 year. However, the differences in germination rates for LN storage periods of 10 minutes to 1 year were not statistically significant.

**Table 2:** **The germination rates of seeds of** ***B. formosana*** **using different periods of cryostorage**

Seeds	Cryopreservation period^1^					
	0 min	10 min	20 min	30 min	1 week	1 Year
TTC staining (%)^2^	77	94	94	93	96	–
Germination rate (%)^3^	79^b^	89^a^	87^a^	89^a^	87^a^	90^a^

^1^Seeds were immersed in loading solution for 30 min and PVS2 for 30 min.

^2^Approximately 300–350 seeds were tested in each TTC-staining.

^3^Values are means of 5 replicates (200–300 seeds per replicate). Means followed by the same letters are not significantly different by Duncan’s test (*P*=0.05). Germination rates were calculated after 4 weeks of culture.

**Figure 2:**
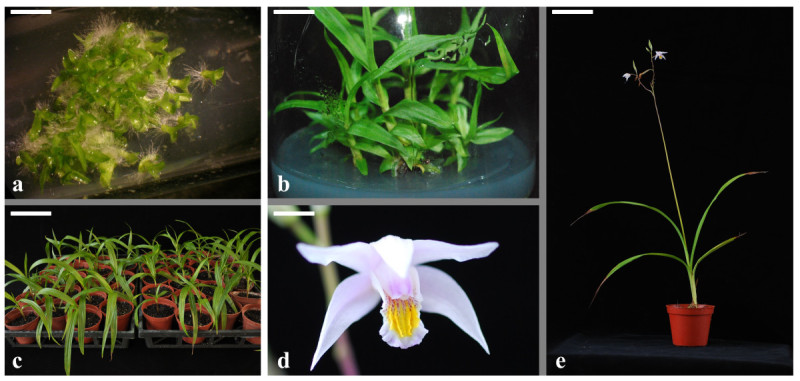
**The** ***B. formosana*** **plants developed from cryopreserved seeds. a**, Germination and plantlet from cryopreserved seeds, 6 weeks after thawing. Before cryopreservation, seeds were cryoprotected with LS for 30 min followed by PVS2 for 30 min (bar = 4.5 mm); **b**, Plantlets from cryopreserved seeds 6 months after sowing *in vitro* (bar = 1.5 cm); **c**, Plantlets growth in greenhouse after 6 month cultivation (bar = 12 cm); **d**, Morphology of flower (bar = 0.6 cm); **e**, Fully developed inflorescence of plantlets (bar = 9.2 cm).

Cryopreserved seeds were cultured on SD medium through three subcultures. At 6 weeks after sowing, germinating seeds turned into green colored, protocorms with leaf and absorbing hairs (Figure 2a), and morphological normalities were observed in all of the plants obtained after 6 months of *in vitro* culture (Figure 2b). The healthy seedlings were transplanted within 6 months after grown in the greenhouse, the plants looked normal morphology (Figure 2c). After 18 months of cultivation, 31 of 70 plants were flowering, and grew normal (Figure 2d and 2e).

## Discussion

The beneficial effects of loading solution pretreatment in cryopreservation methods have been reported for numerous plant species (Takagi, 2000). Loading minimizes injurious membrane changes resulting from severe dehydration with PVS2, and is applied in many of plant vitrification procedures (Ishikawa et al., 1997; Kim et al., 2009). LS with high concentrations of sucrose were reported to increase the survival of cryopreserved orchid seeds (Ishikawa et al., 1997; Hirano et al., [Bibr CR9][Bibr CR10][Bibr CR8]). Our results show that, when PVS2 combined with LS treatments, germination rates were enhanced (Table 1). It is also important to decrease the deleterious effects of PVS2 treatment and enhance the acute dehydration tolerance of seeds. Different periods of loading treatment did not have a significant influence on germination rates (Table 1). However, the seeds lost viability after treated with LS 30 min and without PVS2 dehydration treatment. We conjectured that loading solution raise seeds moisture content and leading to seeds didn’t survive after cryostorage. An appropriate LS is crucial for plant species and explants that are sensitive to PVS2. Our data show a loading time of 10 minutes was optimal. The small diameter of the orchid seeds may contribute to this relatively short treatment period.

Vitrification is a simple, low cost, and practical method for long term preservation of orchid seeds (Thammasiri, 2000). Hirano et al. (2009) reported that *Phaius tankervilleae* survival rates decreased as a result of PVS2 treatment, suggesting that the seeds were damaged by the cryoprotectant. However, seed moisture content can affect viability and germination following cryopreservation because it must be low enough to minimize ice-crystal formation and high enough to avoid desiccation damage (Ozden-Tokatli et al., 2007). In addition, concentrated PVS2 is toxic to seed embryos. Therefore, it is essential to optimize the PVS2-dehydration procedure to prevent damage to the seed embryo caused by chemical toxicity and sudden osmotic stress (Panis et al., 2001). For the Thai orchid *Doritis pulcherrima*, it was shown that seeds exposed to PVS2 solution for 50 min had the highest germination rate, with longer or shorter exposure times resulting in a decrease in survival (Thammasiri, 2000). In a similar study of *B. striata*, embryos precultured in 0.3 M sucrose for 3 days, produced the highest survival when dehydrated in PVS2 for 3 h at 0°C prior to storage in LN (Hirano et al., [Bibr CR9][Bibr CR10]). In our study, seeds that were treated with PVS2 for 10 min prior to storage in LN, showed a significantly higher germination rate (78%) than that observed for PVS2 treatments of 20 min to 120 min (Table 1). Our results also showed that seeds did not survive storage in LN without cryoprotection. According to previous studies, an optimal PVS2 exposure times of 30 minutes to several hours have been reported for the cryopreservation of orchid seeds using vitrification procedures (Ishikawa, 1997; Thammasiri, 2000; Hirano et al. [Bibr CR9][Bibr CR10]; Thammasiri and Soamkul, 2007; Vendrame et al., 2007). Our data show that a shorter PVS2-exposure time (10 min) resulted higher viability in *B. formosana* that were cryopreserved using vitrification. The shorter PVS2-exposure time likely contributed to viability by reducing potential damage due to chemical toxicity or/and osmotic stress.

The TTC test has been previously used for fast and early evaluation of orchid seed viability (Van Waes and Debergh [Bibr CR36][Bibr CR37]; Lauzer et al., 1994). The test result is determined by the appearance of a red color after soaking the seeds in the TTC solution. In our study, seed viabilities assessed by TTC staining were higher than the observed germination rates (Tables 1 and 2). Thus, viability testing based on TTC staining was not an accurate predictor of germination for *B. formosana* after cryopreservation. Rasmussen (1995) reported that viability testing using TTC staining does not necessarily correlate well with the germination rates of orchid seeds. In our study, we propose that the split in the seed coat may have lead to an overestimation of seed viability because the split may have allowed TTC to penetrate the seed coat more easily.

Regardless of the cryopreservation technique used, storage at ultralow temperature under optimal conditions only slighted affected seed germination rate of many species (Pritchard, 1984; Wang et al., 1998; Wood et al., 2000; Popova et al., 2003; Ozden-Tokatli et al., 2007; Hirano et al., 2009; Voronkova and Kholina, 2010). In the literature, cryostorage has been shown to increase germination rates of orchid seeds, compared with other storage methods (Nikishina et al. [Bibr CR17][Bibr CR18][Bibr CR19]; Popov et al., 2004). The germination rate of the seeds from LN treatment in our study was significantly higher (86.5-90.1%) than non-LN treatments (79%, Table 2). As both Nikishina et al. (2001a) and Popova et al. (2003) suggested, the effect of ultralow temperature followed by thawing can cause damage to the seed coat. The culture medium may reach the embryo more easily through the damage seed coat, thereby enhancing germination rates.

Seed banks have traditionally been used for germplasm conservation. However, seed banks often used storage conditions that may cause some seeds to lose viability, as was reported in a previous study of *B. formosana* in which the germination rate decreased after 6 months storage at 3°C (Chang et al., 2006). Thus, cryopreservation provides an effective long-term storage method for the conservation of plant genetic resources because it pauses essentially all biological processes (Gonzalez-Benito et al., 1998b; Benson, 1999). Previous studies of other species have also shown that cryopreservation periods did not affect germination rates (Gonzalez-Benito et al., [Bibr CR6][Bibr CR7]; Pence, 2003). Our results also indicated cryostorage times from 10 min to 1 year did not result in significant variation in germination rates (Table 2), and our method of vitrification and cryopreservation was also effective for maintaining both seed viability and germination rates for all storage times longer than 10 min. However, Walter et al. (2004) refuted the commonly held idea that all biological activity ceases at ultralow temperatures, and proposed that such activity is not only factor that contributes to seed deterioration. Therefore, measurements of seed viability after long term (several decades) cryostorage remains necessary to ensure germplasm survival.

Cryopreservation imposes a series of stresses to plant materials. It is thus necessary to verify that the genetic stability of the cryopreserved material is not altered before routinely using such techniques for long term conservation of plant genetic resources (Engelmann, 2011). Recent studies have been performed that compared the seedling morphology of different orchid species cultured from non-cryopreserved and cryopreserved seeds. Among these species, *D. pulcherrima* (Thammasiri, 2000), *B. striata* (Ishikawa, 1997), *Vanda coerulea* (Thammasiri et al., 2007), *Dendrobium* hybrid (Vendrame et al., 2007), and *P. tankervilleae* (Hirano et al., 2009) did not display significantly different vegetative characteristics. Hirano et al. ([Bibr CR9][Bibr CR10]) showed that *B. striata* plantlets developed from cryopreserved seeds produced normal flowers. In our study, the morphology of both vegetative and reproductive organs of *B. formosana* obtained from cryopreserved seeds was normal with no significant variation observed (Figure 2). Plants derived from cryopreserved seeds were fertile, and the capsules were harvested after manual pollination. New generation plantlets exhibited similar morphology and levels of viability to seeds of plants grown from non-cryopreserved seeds (data not shown). Our experiments have demonstrated that no morphological variation was observed as a result of our method of cryopreservation by vitrification.

## Conclusion

In conclusion, we have shown that *B. formosana* seeds can be preserved for long periods by using cryopreservation and vitrification, and that the vitrification method used in this study is an efficient means by which to preserve orchid seeds that are difficult to preserve under dry and low-temperature conditions. When seeds are treated with LS and PVS2 the germination percentage of *B. formosana* seeds during cryopreservation period was enhanced.

## Authors’ original submitted files for images

Below are the links to the authors’ original submitted files for images. Authors’ original file for figure 1 Authors’ original file for figure 2

## Abbreviations

LS: loading solution
PVS2: Plant vitrification solution 2
TTC: 2,3,5-triphenyltetrazolium chloride
LN: Liquid nitrogen.

## References

[CR1] Benson EE, Benson EE (1999). Cryopreservation. Plant Conservation Biotechnology.

[CR2] Chang C, Chen CT, Tsai TC, Chang WC (2000). A tissue culture protocol for propagation a rare plant species *Lilium speciosum* Thunb var. *gloriosoides* Baker. Bot Bull Acad Sin.

[CR3] Chang C, Sung PG, Chang CH, Chen YC, Lin YH (2006). Seed development and storage of *Bletilla formosana* (Hayata) Schltr. Seed & Nursery Taiwan.

[CR4] Chen YC, Lee TH, Hung HC, Chang C, Chang LZ, Wei FM (2009). The development, cultivation and chemical constituents in pseudobulbs of *Bletilla formosana* (Hayata) Schltr. Bull Taichung District Agr Res Ext Sta.

[CR5] Engelmann F (2011). Use of biotechnologies for the conservation of plant biodiversity. In Vitro Cell Dev Biol Plant.

[CR6] Gonzalez-Benito ME, Fernandez-Llorente F, Perez-Garcia F (1998). Interaction between cryopreservation, rewarming rate and seed humidifcation on the germination of two Spanish endemic species. Ann Bot.

[CR7] Gonzalez-Benito ME, Iriondo JM, Perez-Garica F (1998). Seed cryopreservation: an alternative method for the conservation of Spanish endemics. Seed Sci Technol.

[CR8] Hirano T, Godo T, Miyoshi K, Ishikawa K, Ishikawa M, Mii M (2009). Cryopreservation and low-temperature storage of seeds of *Phaius tankervilleae*. Plant Biotechnol Rep.

[CR9] Hirano T, Godo T, Mii M, Ishikawa K (2005). Cryopreservation of immature seeds of *Bletilla striata* by vitrification. Plant Cell Rep.

[CR10] Hirano T, Ishikawa K, Mii M (2005). Cryopreservation of immature seeds of *Ponerorchis graminifolia* var. *suzukiana* by vitrification. Cryo-Letters.

[CR11] Ishikawa K, Harata K, Mii M, Sakai A, Yoshimatsu K, Shimonura K (1997). Cryopreservation of zygotic embryos of a Japanese terrestrial orchid (*Bletilla striata*) by vitrification. Plant Cell Rep.

[CR12] Kim HH, Lee YG, Park SU, Lee SC, Baek HJ, Cho EG, Engelmann F (2009). Development of alternative loading solutions in droplet-vitrification procedures. Cryo-Letters.

[CR13] Lauzer D, St-arnaud M, Barabe D (1994). Tetrazolium staining and *in vitro* germination of mature seeds of *Cypripedium acaule* (Orchidaceae). Lindleyana.

[CR14] Lin YJ, Chen CC, Yeh FT, Chiu NY, Tsay HS (1994). Tissue culture of *Bletilla formosana* I. The influence of seed maturity and pretreatment on seed germination and seedling development. J. Agr. Res. China.

[CR15] Matsumoto T, Sakai A, Yamada K (1994). Cryopreservation of *in vitro*-grown apical meristems of wasabi (*Wasabia japonica*) by vitrification and subsequent high plant regeneration. Plant Cell Rep.

[CR16] Murashige T, Skoog F (1962). A revised medium for rapid growth and bioassays with tobacco tissue cultures. Physiol Plant.

[CR17] Nikishina TV, Popov AS, Kolomeitseva GL, Golovkin BN (2001). Effect of cryopreservation on seed germination of rate tropical orchids. Russ J Plant Physiol.

[CR18] Nikishina TV, Popov AS, Kolomeitseva GL, Golovkin BN (2001). Cryopreservation of seed of some tropical orchids. Dokl Biochem Biophys.

[CR19] Nikishina TV, Popova EV, Vakhrameeva MG, Varlygina TI, Kolomeitseva GL, Burov AV, Popovich EA, Shirokov AI, Shumilov VY, Popov AS (2007). Cryopreservation of seeds and protocorms of rare temperate orchids. Russ J Plant Physiol.

[CR20] Nitzsche W, Evans DA, Sharp WR, Ammirato PV, Yamada Y (1983). Germplasm preservation. Handbook of Plant Cell Culture, vol 1.

[CR21] Ozden-Tokatli Y, Ozudogru EA, Gumusel F, Lambardi M (2007). Cryopreservation of *Pistacia* spp. seeds by dehydration and one-step freezing. Cryo-Letters.

[CR22] Panis B, Swennen R, Engelmann F (2001). Cryopreservation of plant germplasm. Acta Hort.

[CR23] Pence V, Smith RD, Dickie JB, Linington SH, Pritchard HW, Probert RJ (2003). *In vitro* growth of embryo axes after long-term storage in liquid nitrogen. Seed conservation: turning science into practice.

[CR24] Popov AS, Popova EV, Nikishina TV, Kolomeitseva GL (2004). The development of juvenile plants of the hybrid orchid *Bratonia* after seed cryopreservation. Cryo-Letters.

[CR25] Popova EV, Nikishina TV, Kolometseva GL, Popov AS (2003). The effect of seed cryopreservation on the development of protocorms by the hybrid orchid *Bratonia*. Russ J Plant Physiol.

[CR26] Pritchard HW (1984). LN preservation of terrestrial & epiphytic orchid seed. Cryo-Letters.

[CR27] Rasmussen HN, Rasmussen HN (1995). Cultivation of immature seed. Terrestrial orchids from seed to mycotrophyic plant.

[CR28] Rick CM, Ammirato PA, Evans DA, Sharp WR, Yamada Y (1984). Plant germplasm resources. Handbook of Plant Cell Culture, vol 2.

[CR29] Sakai A, Kobayashi S, Oiyama I (1990). Cryopreservation of nucellar cells of navel orange (*Citrus sinensis* Osb. var. *brasiliensis* Tanaka) by vitrification. Plant Cell Rep.

[CR30] Stanilova MI, Ilcheva VP, Zagorska NA (1994). Morphogenetic potential and *in vitro* micropropagation of endangered plant species *Leucojum aestivum* L. and *Lilium rhodopaeum* Delip. Plant Cell Rep.

[CR31] Su HJ, Huang TC (2000). *Bletilla formosana* (Hayata) Schltr. Flora of Taiwan, vol 5.

[CR32] Takagi H, Engelman F, Takagi H (2000). Recent developments in cryopreservation of hoot apices of tropical species. Cryopreservation of Tropical Germplasm.

[CR33] Tsai SF, Yeh SD, Chan CF, Liaw SI (2009). High-efficiency vitrification protocols for cryopreservation of *in vitro* grown shoot tips of transgenic papaya lines. Plant Cell Tissue Org Cult.

[CR34] Thammasiri K (2000). Cryopreservation of seeds of a Thai orchid (*Doritis pulcherrima* Lindl.) by vitrification. Cryo-Letters.

[CR35] Thammasiri K, Soamkul L (2007). Cryopreservation of *Vanda coerulea* Griff. ex Lindl. seeds by vitrification. ScienceAsia.

[CR36] van Waes JM, Debergh PC (1986). Adaptation of the tetrazolium method for testing the seed viability, and scanning electron microscopy study of some Western European orchids. Physiol Plant.

[CR37] van Waes JM, Debergh PC (1986). *In vitro* germination of some Western European orchids. Physiol Plant.

[CR38] Vendrame WA, Carvalho VS, Maguire I, Dias JMM (2007). *In vitro* germination and seedling development of cryopreservation *Dendrobium* hybrid mature seeds. Sci Hort.

[CR39] Voronkova NM, Kholina AB (2010). Conservation of endemic species from the Russian far east using seed cryopreservation. Biol Bull.

[CR40] Walter C, Wheeler L, Standwood PC (2004). Longevity of cryogenically stored seeds. Cryobiology.

[CR41] Wang JH, Ge JG, Liu F, Bian HW, Huang CN (1998). Cryopreservation of seeds and protocorms of *Dendrobium candidum*. Cryo-Letters.

[CR42] Wood CB, Pritchard HW, Miller AP (2000). Simultaneous preservation of orchid seed and its fungal symbiont using encapsulation-dehydration is dependent on moisture content and storage temperature. Cryo-Letters.

