# Supplementary figures and images for: Cryopreservation the seeds of a Taiwanese terrestrial orchid, Bletilla formosana (Hayata) Schltr. by vitrification
Source: Bot Stud. 2013 Sep 12;54:33. doi: 10.1186/1999-3110-54-33 (PMC5432767; doi:10.1186/1999-3110-54-33)

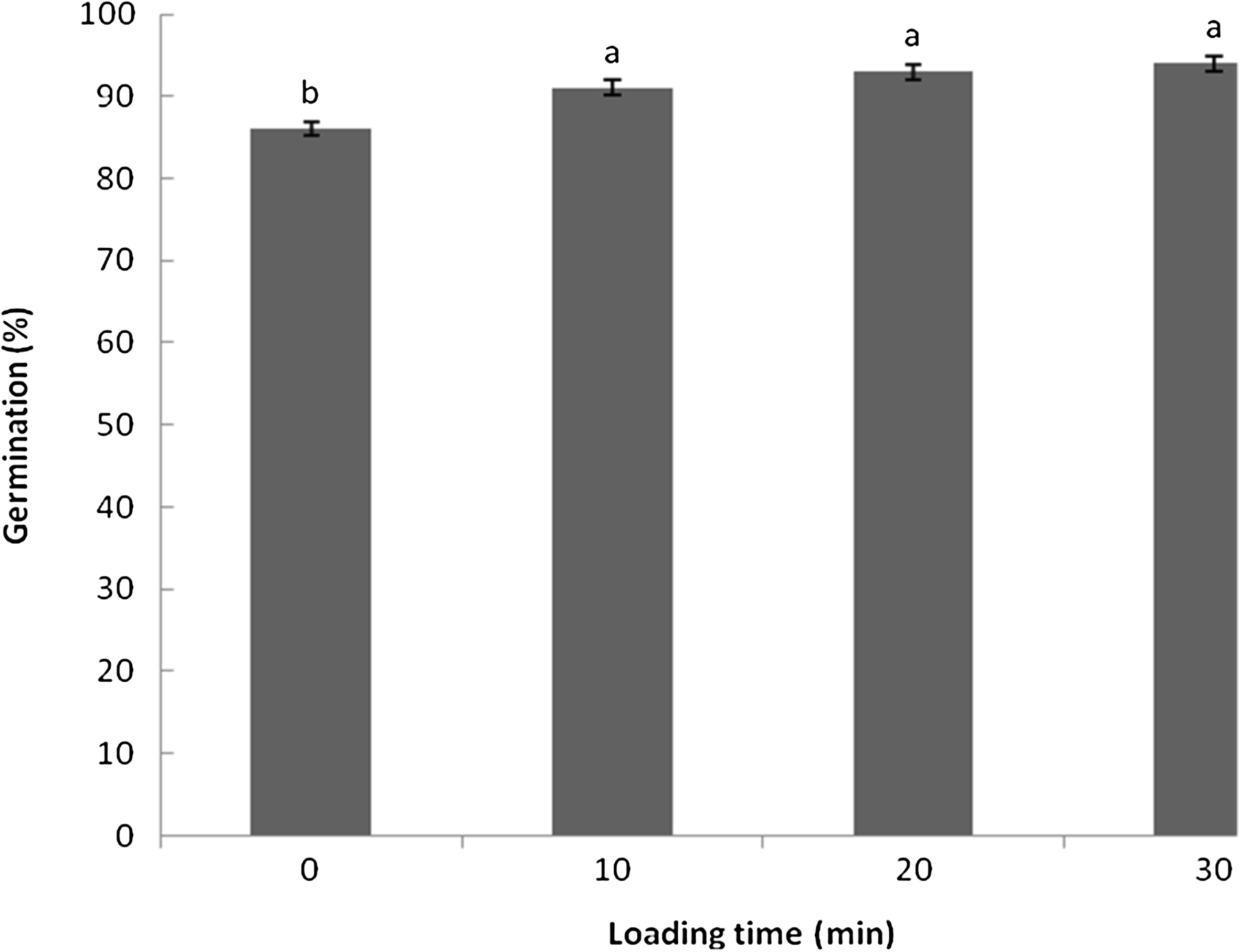

Supplement: Supplementary file 1 — Authors’ original file for figure 1 [file 40529_2012_36_MOESM1_ESM.tif]

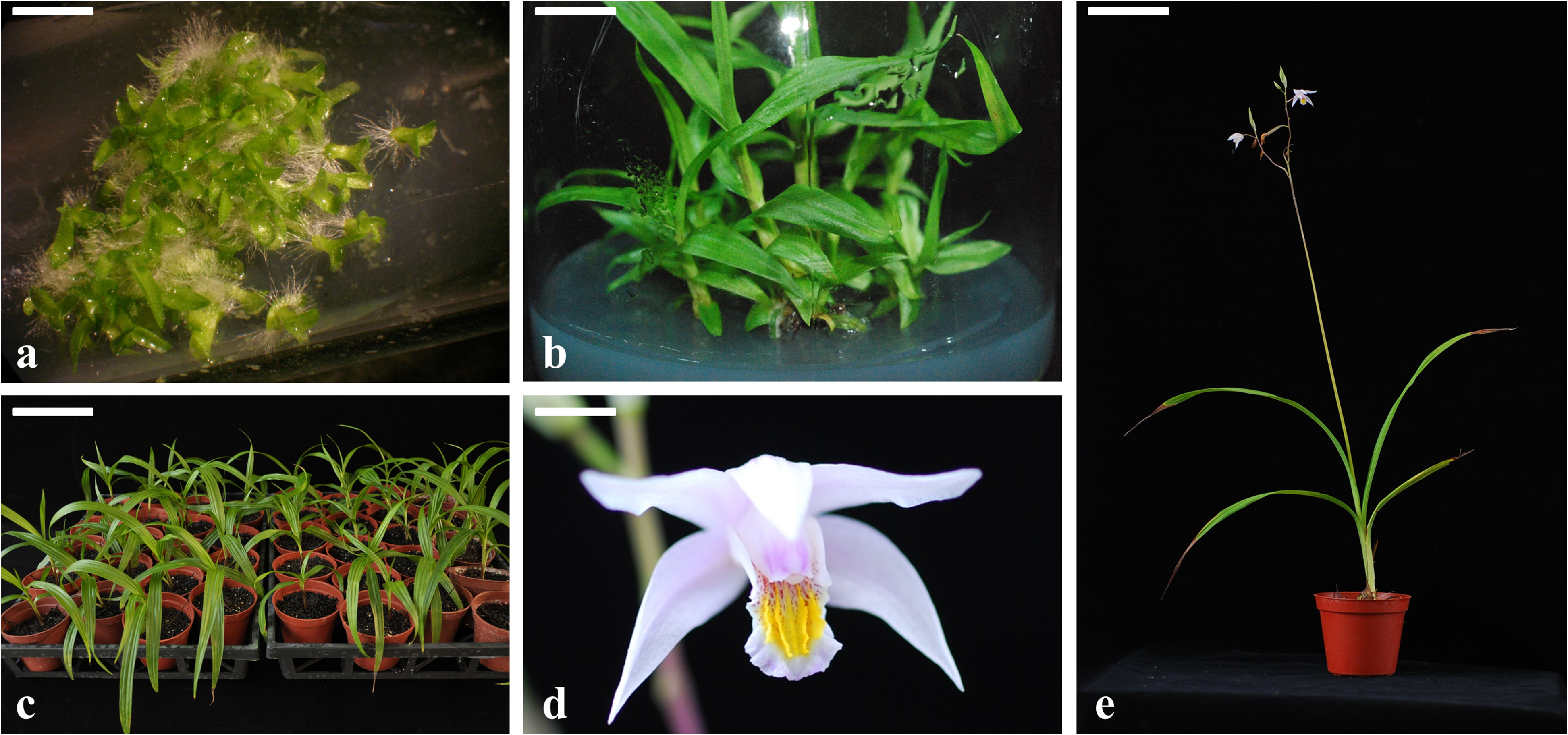

Supplement: Supplementary file 2 — Authors’ original file for figure 2 [file 40529_2012_36_MOESM2_ESM.tif]
